# Quinic acid regulated TMA/TMAO-related lipid metabolism and vascular endothelial function through gut microbiota to inhibit atherosclerotic

**DOI:** 10.1186/s12967-024-05120-y

**Published:** 2024-04-15

**Authors:** Qiao Jin, Chiyuan Zhang, Ran Chen, Luping Jiang, Hongli Li, Pengcui Wu, Liang Li

**Affiliations:** 1 Department of Cardiovascular Medicine, Hengyang Medical School, The Changsha central Affiliated Hospital, University of South China, Changsha, Hunan 410004 China; 2 Department of Cardiovascular Medicine, The Third Xiangya Hospital of Central South University, Changsha, Hunan Province 410013 China; 3 Department of Cardiovascular Medicine, Xiangya Hospital, Central South University, Changsha, Hunan China; 4 Department of Hematology, Xiangya Hospital, Central South University, Changsha, Hunan 410000 China

**Keywords:** Quinic acid, TMAO, Gut microbiota, Metabolism, Atherosclerosis

## Abstract

**Background:**

Quinic acid (QA) and its derivatives have good lipid-lowering and hepatoprotective functions, but their role in atherosclerosis remains unknown. This study attempted to investigate the mechanism of QA on atherogenesis in Apoe−/− mice induced by HFD.

**Methods:**

HE staining and oil red O staining were used to observe the pathology. The PCSK9, Mac-3 and SM22a expressions were detected by IHC. Cholesterol, HMGB1, TIMP-1 and CXCL13 levels were measured by biochemical and ELISA. Lipid metabolism and the HMGB1-SREBP2-SR-BI pathway were detected by PCR and WB. 16 S and metabolomics were used to detect gut microbiota and serum metabolites.

**Results:**

QA or low-frequency ABX inhibited weight gain and aortic tissue atherogenesis in HFD-induced Apoe−/− mice. QA inhibited the increase of cholesterol, TMA, TMAO, CXCL13, TIMP-1 and HMGB1 levels in peripheral blood of Apoe−/− mice induced by HFD. Meanwhile, QA or low-frequency ABX treatment inhibited the expression of CAV-1, ABCA1, Mac-3 and SM22α, and promoted the expression of SREBP-1 and LXR in the vascular tissues of HFD-induced Apoe−/− mice. QA reduced *Streptococcus_danieliae* abundance, and promoted *Lactobacillus_intestinalis* and *Ileibacterium_valens* abundance in HFD-induced Apoe−/− mice. QA altered serum galactose metabolism, promoted SREBP-2 and LDLR, inhibited IDOL, FMO3 and PCSK9 expression in liver of HFD-induced Apoe−/− mice. The combined treatment of QA and low-frequency ABX regulated microbe-related Glycoursodeoxycholic acid and GLYCOCHENODEOXYCHOLATE metabolism in HFD-induced Apoe−/− mice. QA inhibited TMAO or LDL-induced HCAECs damage and HMGB1/SREBP2 axis dysfunction, which was reversed by HMGB1 overexpression.

**Conclusions:**

QA regulated the gut-liver lipid metabolism and chronic vascular inflammation of TMA/TMAO through gut microbiota to inhibit the atherogenesis in Apoe−/− mice, and the mechanism may be related to the HMGB1/SREBP2 pathway.

**Supplementary Information:**

The online version contains supplementary material available at 10.1186/s12967-024-05120-y.

## Introduction

Currently, atherosclerotic diseases, such as myocardial infarction and stroke, are responsible for the majority of deaths worldwide [1]. It is widely believed that atherosclerosis is a chronic inflammation of the blood vessels caused by the interaction of these risk factors (Hypercholesterolemia, hypertension, diabetes, smoking, etc.) and cells in the artery wall [2]. Recently, there is increasing evidence that a large number of metabolites derived from the gut microbiome are responsible for atherosclerotic events [3]. Gut microbiome metabolism of dietary choline may promote atherosclerosis through trimethylamine (TMA), which is rapidly absorbed in the liver and converted to the atherogenic trimethylamine-n-oxide (TMAO) [4]. The presence and expression of the CutC gene in the choline TMA lyase pathway of the bacterial community was sufficient to enhance the platelet reactivity and thrombogenic potential of the host, which can be used as a molecular target for the treatment of atherosclerosis [5]. Modulating TMAO production and the associated gut microbiome may be a promising strategy for anti-atherosclerosis therapy.

Quinic acid (QA), a metabolite of chlorogenic acid, is particularly abundant in dietary sources and may reduce the risk of type 2 diabetes and cardiovascular disease [6]. 5-caffeyl-QA inhibited HFD-induced increases in body weight, visceral fat pad weight, lipid levels, and serum and liver free fatty acids in a dose-dependent manner [7]. QA was found to be an active component in the resistance to *Pseudomonas aeruginosa* biofilm in *Lonicerae Japonicae Flos*, suggesting that QA may have potential as an antibacterial film agent against *Pseudomonas aeruginosa* and associated infections [8]. Acyl-QA derivatives from *Carissa spinarum* were known to have good antioxidant and liver protective activities [9]. QA derivatives from *Erycibe obtusifolia* have antioxidant and tyrosinase inhibitory activities [10]. However, the role of dietary QA as a nutritional supplement in atherosclerosis remains unknown. Therefore, exploring the role of QA in atherosclerosis might help develop new drugs.

Currently, QA has been shown to inhibit TNF-α-induced CD106 expression by inhibiting the MAP kinase and NF-κB signaling pathways and the adhesion of vascular smooth muscle cell (VSMC), which could explain QA’s ability to inhibit vascular inflammation, such as atherosclerosis [11]. QA derivatives also demonstrated in vitro antimicrobial activity, antioxidant activity against DPPH (1, 1-DIPHENYL − 2-picrylhydrazyl) free radicals and LPS-induced neuroinflammatory activity [12, 13]. As *Erigeron annuus Pers* water extract (EAW), QA plays an anti-obesity role in HFD-induced obese mice [14]. D- (-) -QA showed a significant lipid-lowering activity by reducing the increase of cholesterol, triglycerides, LDL, VLDL, and other liver parameters (such as SGOT and SGPT) in the serum of hyperlipidemia mice induced by a high fat diet [15]. Therefore, we sought to explore the potential mechanism of QA in gut microbiota TMA metabolism promoting atherosclerosis, with a view to providing new insights into the treatment of atherosclerosis.

## Materials and methods

### Animal testing and grouping

Thirty C57BL/6J Apoe−/− mice were purchased from Hunan Slyke Jingda Laboratory Animal Co., LTD. The mice were randomly divided into NC group, HFD group, HFD + QA group, HFD + ABX group, HFD + ABX + QA group, and 6 mice/group. Mice in NC group were given a standard diet. Mice in 2–5 groups were given a HFD to induce atherosclerosis model. QA (10 mg/kg) was given five times a week intraperitoneal injection [16]. ABX (0.5 g/L vancomycin, 1 g/L neomycin sulfate, 1 g/L metronidazole, 1 g/L ampicillin) were given in their drinking water every two weeks for three days [17, 18]. After 12 weeks, fecal samples, peripheral blood serum, liver and aortic tissues were collected for further analysis.

This study was approved by the Ethics Committee of Nanhua University Affiliated Changsha Central Hospital (201,812). All experimental procedures were conducted in accordance with institutional guidelines for the use of experimental animals.

### Cell experiment and grouping

To investigate the cytotoxicity of QA, human coronary artery endothelial cells (HCAECs, HUM-iCell-c006, iCell) were treated with 1, 2.5, 5, 10 and 20 µM QA. CCK-8 was applied to detect the toxicity of QA to HCAECs. To explore the effect of QA on TMAO-induced HCAECs damage, cells were randomly divided into Control, TMAO (200 µmol/L) [19], TMAO + QA groups. To explore the effect of QA on TMAO-induced HCAECs on LDL trans-endocytosis, HCAECs were randomly divided into Control, LDL, LDL + QA, LDL + TMAO, TMAO + LDL + QA, oe-NC, QA + oe-NC, QA + oe-HMGB1 groups. HCAECs was treated with 20 µg/mL Human low-density lipoprotein (LDL, L8292, Sigma-Aldrich) [20] in LDL group. All the cell experiment was repeated three times for each group.

### Oil red O staining

The aorta was transferred from the fixator to the water and clean out the fixator. Fat outside the aortic wall was remove as much as possible using eye tweezers on a microdissection table. The aorta was cut open with ophthalmic shears. The cut aorta was placed in oil red O staining solution (Abiowell, China) and stained for 10 min at room temperature. The aorta was clipped and differentiated in 75% alcohol until there was no obvious red wall and the fat was bright red. The differentiated aorta was clipped into water to wash 75% alcohol. The stained aorta is then stretched open and photographed. Image Pro Plus 6.0 was applied to analyze the cell Oil Red O staining results and calculate the ratio of the plaque area/vascular area. After the photo is taken, the aorta is placed back in the fixator.

### Hematoxylin-eosin (HE) staining

Mouse aortic sinus tissue was fixed and sliced. The slices were roasted at 60℃ for 12 h. The slices are dewaxed to water. The sections were then stained with hematoxylin (Abiowell, China) and eosin (Abiowell, China), respectively. Finally, the sections were sealed and observed under a microscope (BA210T, Motic).

### Immunohistochemistry (IHC)

Heat repair antigen was performed on aorta and liver sections. The sections were added with 1% periodate at room temperature for 10 min to inactivate endogenous enzyme. Sections were added with anti-PCSK9 (55206-1-AP, 1:250, Proteintech, USA), anti-Mac-3 (10397-1-AP, 1:200, Proteintech, USA) and anti-SM22α (10493-1-AP, 1:50, Proteintech, USA), 4℃ overnight. Sections were added with 50 ∼ 100 µL anti-IgG antibody-HRP polymer and incubated at 37℃ for 30 min. The sections were stained by DAB and observed under microscope (BA410T, Motic).

### Biochemical testing

Low density lipoprotein cholesterol (LDL-C, A113-1-1, Microplate method, NJJCBio), triglyceride (TG, A110-1-1, GPO-PAP enzyme method, NJJCBio), High density lipoprotein cholesterol (HDL-C, A112-1-1, Microplate method, NJJCBio) and total cholesterol (T-CHO, A111-1-1, COD-PAP method, NJJCBio) kits were applied to detect LDL-C, TG, HDL-C and T-CHO levels in serum.

### ELISA

High mobility group box chromosomal protein 1 (HMGB1, SEA399Mu, Cloud-Clone Corp), tissue inhibitor of metalloproteinase-1 (TIMP-1, MTM100, Bio-Techne China Co., Ltd.) and chemokine ligand 13 (CXCL13, MCX130, Bio-Techne China Co., Ltd.) kits were applied to detect HMGB1, TIMP-1 and CXCL13 levels.

### CCK-8

CCK-8 (NU679, Tongren, Japan) kit was used to prepare CCK-8 solution (1:10) with complete medium. The 96-well plate was inoculated with 1 × 10^4^ cells/well density. Cells were added to 100 µL of CCK-8-containing medium per well and incubated for 4 h to detect on the Bio-Tek label (MB-530, Huisong).

### LDL uptake

After the above-mentioned grouping of cells, the culture medium was replaced with fresh medium containing 5 µg/mL BODIPY-FL-LDL (L3483, Invitrogen, USA), and incubated for 1 h. The cells were then fixed with 4% paraformaldehyde for 10 min. The cell nucleus was stained with DAPI at a dilution of 1:500. The stained cells were imaged using a fluorescence microscope (DSZ2000X, Cnmicro), and the immunofluorescence intensity was quantified using Image J software.

### RT-qPCR

Total RNA was extracted by Trizol (15,596,026, Thermo). Reverse transcription of cDNA was performed by using mRNA (CW2569, CWBIO, China) kit. The sequences of target genes were searched on NCBI. Primers were designed by primer5 software. Primers were synthesized by Beijing Muscularidae. The target genes were detected by UltraSYBR Mixture (CW2601, CWBIO, China) and fluorescence quantitative RCP instrument (PIKOREAL96, Thermo). β-actin as internal reference. The relative expression of target genes (Table 1) in each individual sample compared to the reference gene was calculated using 2^−ΔΔCt^ algorithm. ANOVA and Tukey’s post-test were used to analyze the differences in gene expressions between groups.

**Table 1:** Primer sequence

Gene	Primer sequence	Length
IDOL	FGAGAAACCGGATCTCCCAGC	216 bp
	RTCTCCAAACTTGGTCTGGGC	
FMO3	FAGGTTACCATGGGGAAGAAAG	137 bp
	RAAATTTCCACAGGCCCCCAA	
SREBP-2	FTGAGCCAGGAAGCCCTCTAT	137 bp
	RGGGGGTTAAAGGAGAGGCAC	
LDLR	FACCAATCTCTAAGCCAAACCC	188 bp
	RCAGATCATTTCCGACGCCAT	
HMGB1	FCTATATTACGGTTTGCCCCTT	209 bp
	RACTGGCACTTTAAGAAAACGAT	
COX-2	FCTCTATCACTGGCATCCCCTT	169 bp
	RCATTCCTACCACCAGCAACCC	
IL-6	FGCAATAACCACCCCTGACCCAA	154 bp
	RGCTACATTTGCCGAAGAGCC	
E-selectin	FTATGGCTGAAACCGCAACACC	140 bp
	RATCCTTTCCCTTCATTAGCCAAC	
ICAM1	FTCTTCCTCGGCCTTCCCATA	152 bp
	RAGGTACCATGGCCCCAAATG	
SRBI	FACTTTCCAGGCATGTTCCCCTT	151 bp
	RTCAACCTTGCTCAGCCCGTTC	
H-actin	F ACCCTGAAGTACCCCATCGAG	224 bp
	R AGCACAGCCTGGATAGCAAC	

### Western blot

Total protein was extracted by radioimmunoprecipitation analysis (RIPA) and lysis buffer. The protein concentration was determined by bicinchoninic acid method. Protein samples were separated by 12% sodium dodecyl sulfate-polyacrylamide gel electrophoresis (SDS-PAGE). The isolated proteins were transferred to a polyvinylidene fluoride film. The membrane was incubated with the first antibody (Table 2) overnight at 4 °C. The membrane was then incubated with goat anti-IgG (H + L) secondary antibody (AWS0001, AWS0002, 1:5000, Abiowell, China) at 37 °C for 90 min. Visualization was performed with ECL Plus hypersensitive luminescence solution (AWB0005, Abiowell, China). Imaging analysis was performed with GE Healthcare software (Life Sciences, USA) to obtain the gray value of proteins. Gray value was used to calculate the intergroup differences in protein expression.

**Table 2:** Antibody information

Proteins	Items	Source	Concentration	Weight	Brand
CAV1	16447-1-AP	Rabbit	1:1000	20-25KD	proteintech
ABCA1	ab18180	Mouse	1:200	254KD	abcam
SREBP1	ab28481	Rabbit	1:2000	68KD	abcam
LXR	ab176323	Rabbit	1:3000	50KD	abcam
IDOL	ab74562	Rabbit	1:1000	50KD	abcam
FMO3	ab126711	Rabbit	1:5000	56KD	abcam
SREBP2	ab30682	Rabbit	1:1000	126KD	abcam
LDLR	ab52818	Rabbit	1:1000	100KD	abcam
P-MAPK14	28796-1-AP	Rabbit	1:1000	38-42KDa	proteintech
P-P65	ab76302	Rabbit	1:1000	65KDa	abcam
ZO-2	ab191133	Rabbit	0.2 µg/mL	131KDa	abcam
VE-cadherin	ab205336	Rabbit	1:1000	90,125KDa	abcam
Occludin	27260-1-AP	Rabbit	1:1000	59KDa	proteintec
HMGB1	ab79823	Rabbit	1:20000	25KDa	abcam
SR-BI	21277-1-AP	Rabbit	1:800	61-82KDa	proteintech
LDL-R	ab52818	Rabbit	1:5000	100KDa	abcam
β-actin	66009-1-Ig	Mouse	1:5000	42KD	proteintech

### 16 S rRNA sequence

DNA was extracted from fecal samples using the fecal genome DNA extraction kit (TIANGEN, #DP328-02). The Phusion enzyme (APExBIO K1031) and bacterial primers for the V3-V4 region of 16S rRNA gene (357F 5’-ACTCCTACGGRAGGCAGCAG-3’ and 806R5’-GGACTACHVGGGTWTCTCATAT-3’) were used for PCR amplification, insertion and library construction. Illumina novaseq6000 PE250 was used for mixed sequencing to obtain raw data. Qiime 2 (2020.2) analysis process was applied to call DADA2 for quality control to get clean data. Species annotations were made for each ASV/OTU sequence using the silva-132-99 database. Qiime 2 software calculates the Alpha and beta diversity indices (Principal component analysis and Anosim analysis) for each sample, as well as the relative abundance of species. Principal component analysis (PCA) analyzes the community distribution of different samples to reflect the similarities and differences between samples [21]. The closer two samples are, the more similar their species composition. R software (VennDiagram package) and jvenn (http://www.bioinformatics.com.cn/static/others/jvenn/example.html) page were used for sample or between groups of common and unique ASVs visualization. LDA Effect Size (LefSe, https://github.com/SegataLab/lefse) was applied to analyze the different microbiota.

### Metabolomics

The 100 uL sample mixture was precipitated with 200 uL methanol. All samples were centrifuged at 4℃, 14,000 g for 10 min. The supernatant was analyzed by UHPLC system (1290, Agilent Technologies) with UPLC BEH column (1.7 μm 2.1 × 100 mm, Waters), TripleTOF 6600 (Q-TOF, AB Sciex) and QTOF 6550 (Agilent). MS raw data files were converted to mzXML format by R package XCMS (version 3.2). A data matrix consisting of retention time (RT), mass/charge ratio (m/z) and peak intensity was generated. MetaboAnalyst platform (https://www.metaboanalyst.ca/) was used for bioinformatics analysis [22]. Statistical analysis (single factor) included multivariate statistics - PCA, partial least squares-discriminant analysis (PLS-DA), etc. PCA uses an “unsupervised mode” to clearly demonstrate the redundancy within data groups and the differences between groups, thereby assessing the replicability of the data. PLS-DA uses partial least squares regression to effectively reduce the number of variables (metabolites) in high-dimensional metabolomics data to assess the relationship between metabolite expression levels and sample categories. The Kyoto Encyclopedia of Genes and Genomes (KEGG, https://www.kegg.jp/) pathway database was used for functional prediction in the MetaboAnalyst platform.

### Correlation analysis between microbiota and metabolites

Based on the Pearson algorithm, we calculated the correlation between microbiota (LDA ≥ 3) and metabolites (*P* < 0.01). Due to missing relative abundance data for the *g_Clostridium_innocuum_group*, we only performed correlation analysis for 31 microbiotas and 13 metabolites. Additionally, a heatmap was used to display the results of the correlation analysis for the 31 microbiota and 13 metabolites.

### Data statistics and analysis

Statistical analysis of data in this study was performed by Graphpad Prism8.0 statistical software. Measurement data are expressed as mean ± standard deviation. First, tests of normality and homogeneity of variance were conducted, which were consistent with normal distribution and homogeneity of variance. Unpaired t test was used for inter-group comparison. One-way ANOVA or ANOVA of repeated measurement data and Tukey’s post-test were used for inter-group comparison. *P* < 0.05 meant the difference was statistically significant.

## Results

### QA inhibited HFD-induced atherogenesis in Apoe−/− mice

**Fig. 1:**
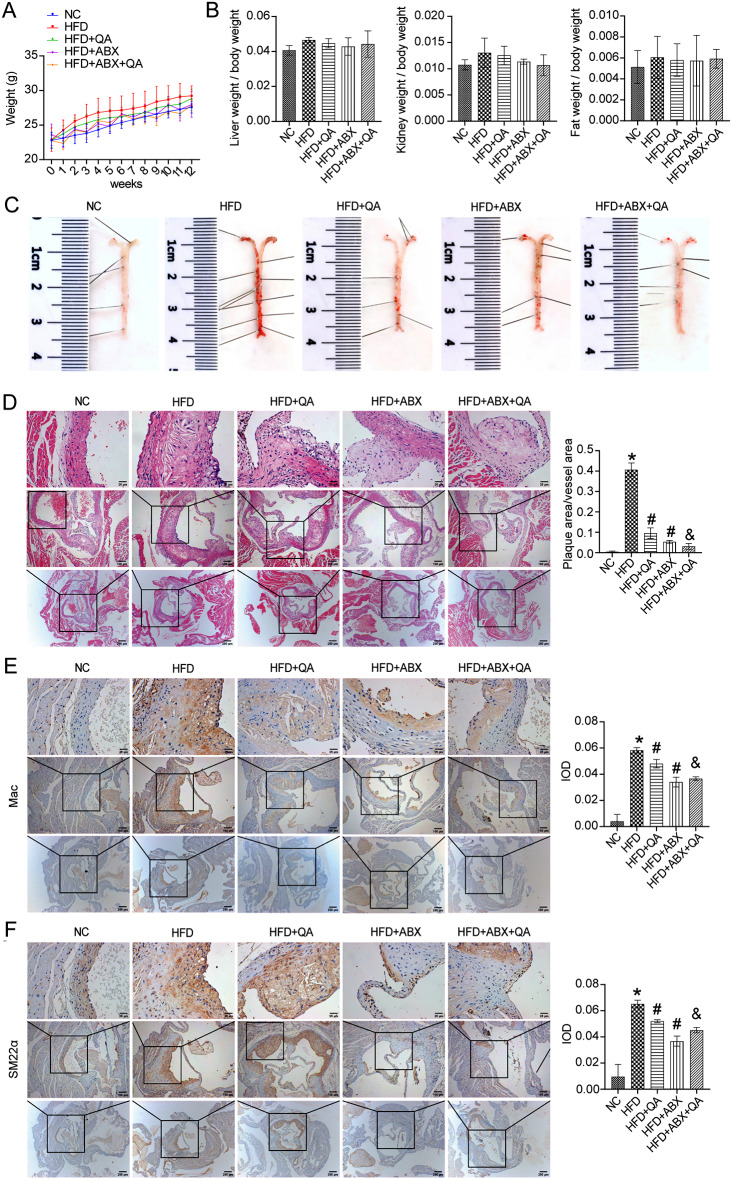
QA improved atherosclerotic disease characterization in HFD-induced Apoe−/− mice. **(A-B)** Changes of body weight, liver/body weight, kidney/body weight, fat/body mass index in mice. **(C-D)** HE and Oil Red O staining were used to observe the pathological changes of vascular plaques. Scale bar = 250, 100, and 40 μm. **(E-F)** The expression of Mac-3 (positive macrophages) and SM22α (positive smooth muscle cells) was investigated by IHC. Scale bar = 250, 100, and 40 μm. *P < 0.05 vs. NC, #*P* < 0.05 vs. HFD, &*P* < 0.05 vs. HFD + QA

We continuously tracked and recorded the changes of mice body weight from 0 ∼ 12 weeks (Fig. 1A). It was observed that the body weight of mice in HFD group was higher and that in NC group was lower at the same time point, but there was no significant difference between the groups (Fig. 1A). At week 12, we measured liver/body weight, kidney/body weight, and fat/body mass index in mice, showing a slight increase in HFD and a decrease in QA or low-frequency ABX intervention, but no significant difference between groups (Fig. 1B). Active vascular tissue Oil Red O staining combined with HE staining showed no significant changes in the NC group (Fig. 1D). In HFD group, there were obvious red atherosclerotic plaques attached to the inner wall of the blood vessels, accompanied by irregular bulges on the surface of the blood vessels, which proved the atherosclerotic lesions of the blood vessels (Fig. 1C-D). QA, ABX, or QA + ABX groups had smooth vascular surface and no obvious red plaque lesions on vascular wall (Fig. 1C-D). Furthermore, quantifying of active vascular tissue oil red O staining showed that compared to the NC group, the plaque area/vascular area significantly increased in the HFD group, but decreased after QA, ABX, or QA + ABX treatment (Fig. 1C-D). Among them, the QA + ABX group had a lower plaque area/vascular area, indicating a better treatment effect (Fig. 1C-D). Compared with the NC group, HFD induced increased expression of Mac-3 and SM22α in the vascular tissues of Apoe−/− mice (Fig. 1E-F), demonstrating the aggregation of macrophages and smooth muscle cells. QA or low-frequency ABX treatment inhibited the increased expression of Mac-3 and SM22α in vascular tissue of HFD-induced Apoe−/− mice (Fig. 1E-F). In addition, the expression of Mac-3 and SM22α in QA + ABX group were lower than that in QA group, but has no significant changes with that in ABX group (Fig. 1E-F), proving that low-frequency ABX treatment may promote the effect of QA. These results suggested that QA inhibited the aggregation of vascular macrophages and smooth muscle cells in Apoe−/− mice induced by HFD, which might be related to lipid endocytosis and atherosclerosis.

### QA improved inflammation and lipid levels in peripheral blood of Apoe−/− mice induced by HFD

**Fig. 2:**
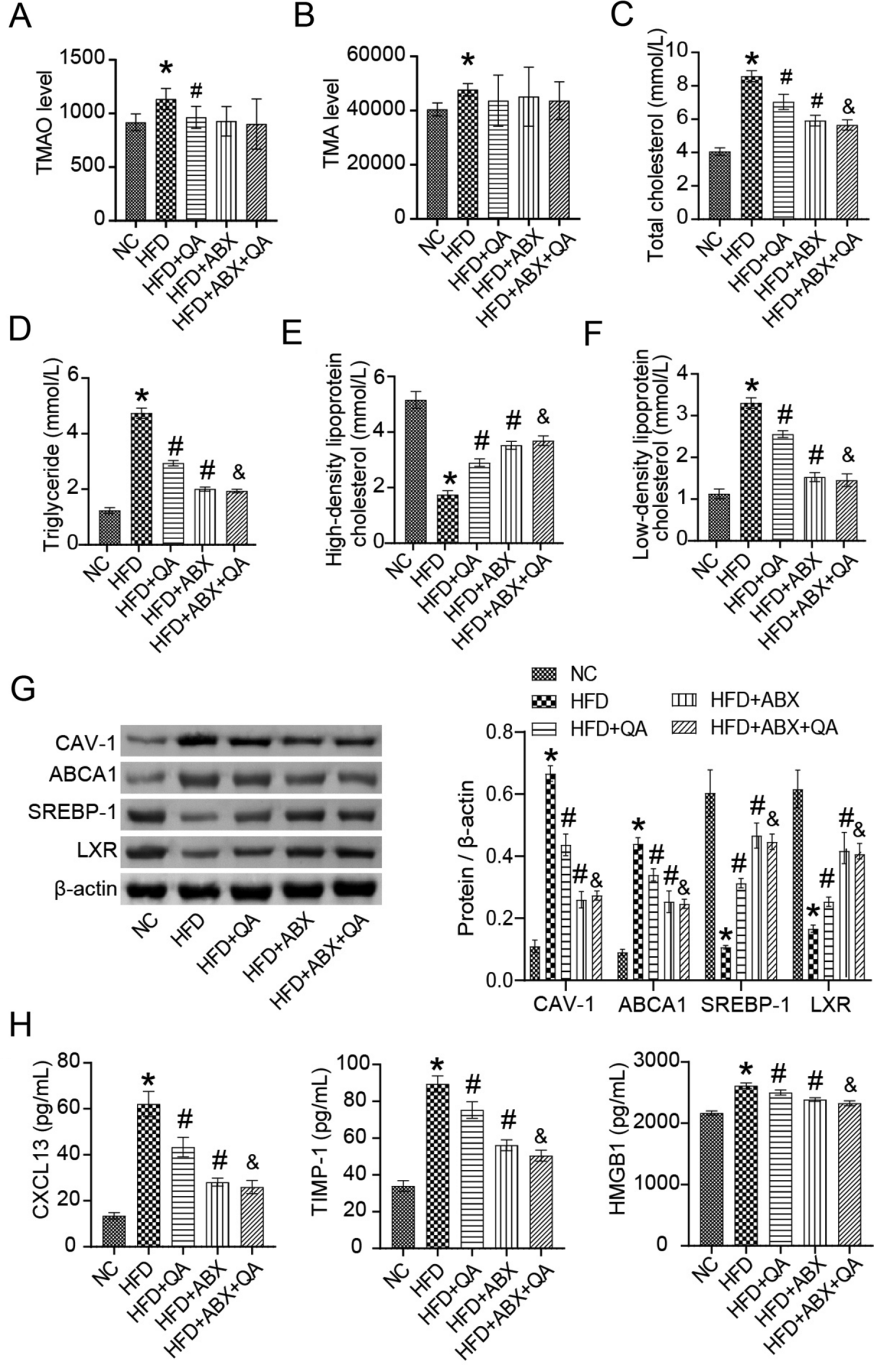
QA improved inflammation and lipid levels in peripheral blood of Apoe−/− mice induced by HFD. **(A-B)** Peripheral blood TMAO and TMA levels were determined by non-targeted metabolism. **(C-F)** The TC, TG, HDL-C and LDL-C were tested by ELISA. **(G)** The CAV-1, ATP binding cassette transporter A1 (ABCA1), sterol regulatory element-binding protein-1 (SREBP-1) and liver-X-receptor (LXR) expression in the vascular tissues were analyzed by western blot. **(H)** ELISA was applied to analyze the CXCL13, TIMP-1 and HMGB1 levels. *P < 0.05 vs. NC, #*P* < 0.05 vs. HFD, &*P* < 0.05 vs. HFD + QA

TMAO and TMA levels in peripheral blood of Apoe−/− mice were significantly increased by HFD, but were down-regulated by QA or low-frequency ABX intervention (Fig. 2A-B). However, there was no significant change in TMAO and TMA levels in the HFD + ABX + QA group compared to the HFD + ABX or HFD + QA groups, possibly due to low-frequency ABX’s treatment of gut microbes (Fig. 2A-B). HFD induced an increase in T-cho, TG and LDL-C levels and a decrease in HDL-C levels in Apoe−/− mice compared with the NC group (Fig. 2C-F). QA or low-frequency ABX intervention reversed HFD-induced changes in cholesterol levels in Apoe−/− mice (Fig. 2C-F). However, there was no significant change in cholesterol levels in the HFD + ABX + QA group compared with the HFD + ABX group, which may be related to the effect of low-frequency ABX on gut microbes (Fig. 2C-F). It is known that lipid transport proteins (CAV-1, ABCA1, SREBP-1, LXR, etc.) and inflammation (HMGB1, CXCL13, TIMP-1, etc.) in blood vessels mediate the formation of atherosclerosis [23–25]. In this study, compared with NC group, HFD induced an increase in the expression of CAV-1 and ABCA1 proteins and a decrease in the expression of SREBP-1 and LXR in the vascular tissues of Apoe−/− mice (Fig. 2G). QA or low-frequency ABX treatment inhibited the expression of CAV-1 and ABCA1 proteins and promoted the expression of SREBP-1 and LXR in the vascular tissues of HFD-induced Apoe−/− mice (Fig. 2G). The results showed that QA inhibited the lipid deposition ability of Apoe−/− mice induced by HFD. Compared with the NC group, HFD induced increased levels of CXCL13, TIMP-1 and HMGB1 in peripheral blood of Apoe−/− mice, indicating the occurrence of chemokines, collagen deposition and excessive inflammation (Fig. 2H). QA or low-frequency ABX treatment inhibited the increase of CXCL13, TIMP-1 and HMGB1 levels in the peripheral blood of Apoe−/− mice induced by HFD (Fig. 2H). However, the CAV-1, ABCA1, SREBP-1, LXR, CXCL13, TIMP-1 and HMGB1 levels were lower in the HFD + ABX + QA group compared with the HFD + QA group (Fig. 2G-H), which proved that low-frequency ABX treatment may promote the effect of QA. QA inhibited peripheral blood inflammation and vascular lipid levels in Apoe−/− mice induced by HFD.

### QA regulated the gut microflora structure of Apoe−/− mice induced by HFD

**Fig. 3:**
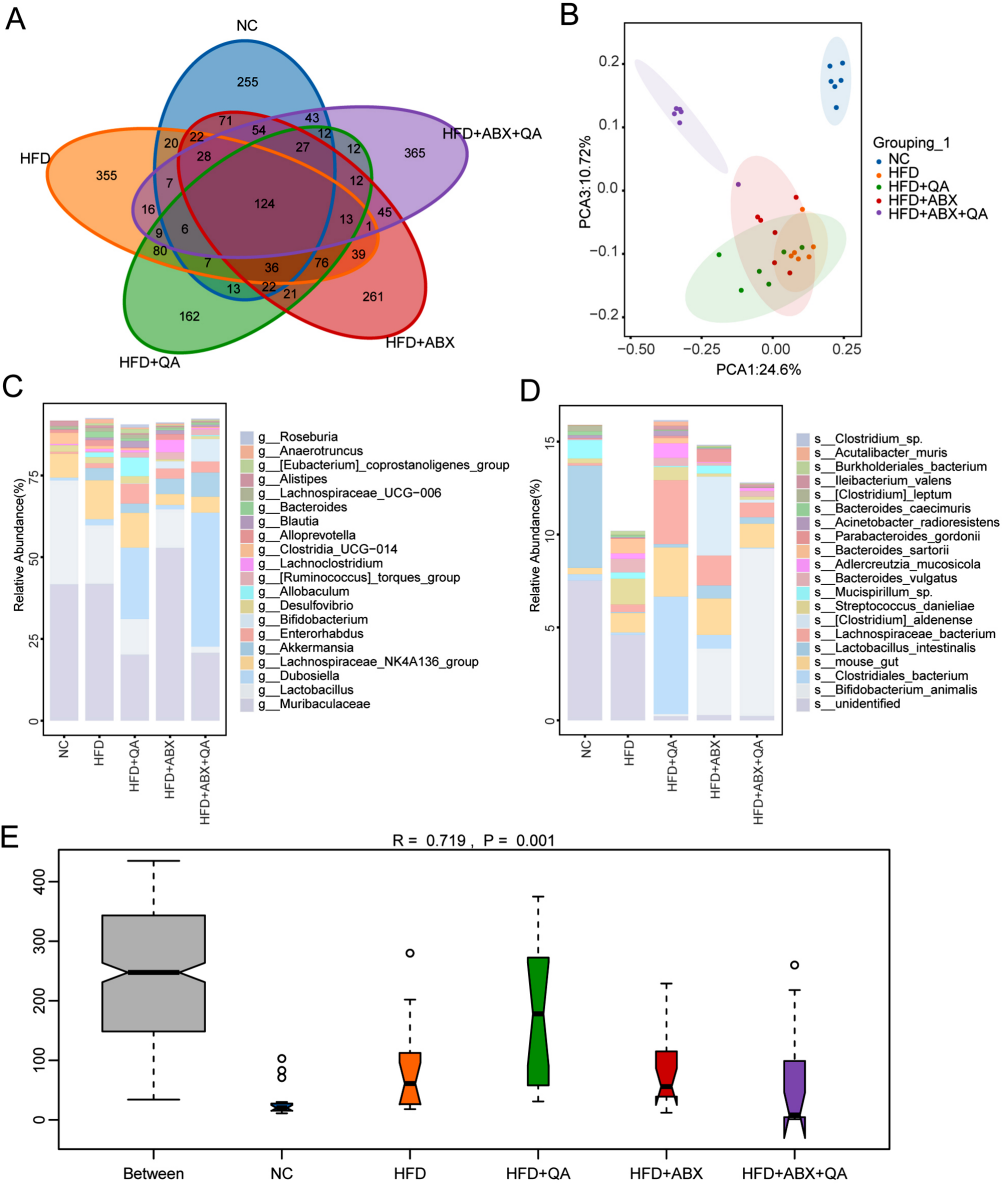
QA improved the intestinal microflora composition of Apoe−/− mice induced by HFD. **(A)** Venn diagram. **(B)** The sample similarity was analyzed by PCA. **(C-D)** The microbiota abundance at genus and species levels. **(E)** Anosim analysis

HFD induced an increase in the number of gut microflora in Apoe−/− mice, but decreased after QA or low-frequency ABX intervention (Fig. 3A). Compared with QA group, ABX combined with QA intervention increased the number of gut microflora in Apoe−/− mice induced by HFD (Fig. 3A). PCA analysis showed that there was crossover of samples in HFD, HFD + QA and HFD + ABX groups, but there was deviation between NC and HFD + ABX + QA groups (Fig. 3B). The results suggested that HFD induced changes in intestinal flora composition, and ABX + QA combined intervention may reconstruct intestinal flora composition (Fig. 3B). Species annotation analysis showed that genus level microorganisms were mainly composed of *Muribaculaceae*, *Lactobacillus*, *Dubosiella*, *Lachnospiraceae_NK4A136_group*, *Akkermansia*, *Enterorhabdus*, *Bifidobacterium*, *Desulfovibrio*, *Allobaculum*, *[Ruminococcus]_torques_group*, *Lachnoclostridium*, *Clostridia_UCG-014*, *Alloprevotella*, *Blautia*, *Bacteroides*, *Lachnospiraceae_UCG-006*, *Alistipes*. *[Eubacterium]_coprostanoligenes_group*, *Anaerotruncus* and *Roseburia* (Fig. 3C). Anosim analysis showed significant differences between groups (*R* = 0.719, *P* = 0.001) (Fig. 3E). Species level abundance analysis showed *Bacteroides_caecimuris* and *Lactobacillus_intestinalis* were prevalent in NC group (Fig. 3D and Supplementary Fig. 1). *Streptococcus_danieliae*, *Bacteroides_sartorii*, *Burkholderiales_bacterium*, *Adlercreutzia_muris* and *Firmicutes_bacterium* were prevalent in HFD group (Fig. 3D and Supplementary Fig. 1). *Adlercreutzia_mucosicola*, *Acutalibacter_muris* and *Ileibacterium_valens* were prevalent in QA group (Fig. 3D and Supplementary Fig. 1). *Clostridium__aldenense*, *Parabacteroides_gordonii*, *Bacteroides_stercorirosoris*, *Parabacteroides_goldsteinii*, *Cuneatibacter_caecimuris* and *Streptococcus_hyointestinalis* were prevalent in ABX group (Fig. 3D and Supplementary Fig. 1). *Bifidobacterium_animalis* was prevalent in ABX + QA group (Fig. 3D and Supplementary Fig. 1). These results indicated that QA could change the number and abundance of gut microflora in Apoe−/− mice induced by HFD.

### QA regulated the abundance of gut microbiota in Apoe−/− mice induced by HFD

**Fig. 4:**
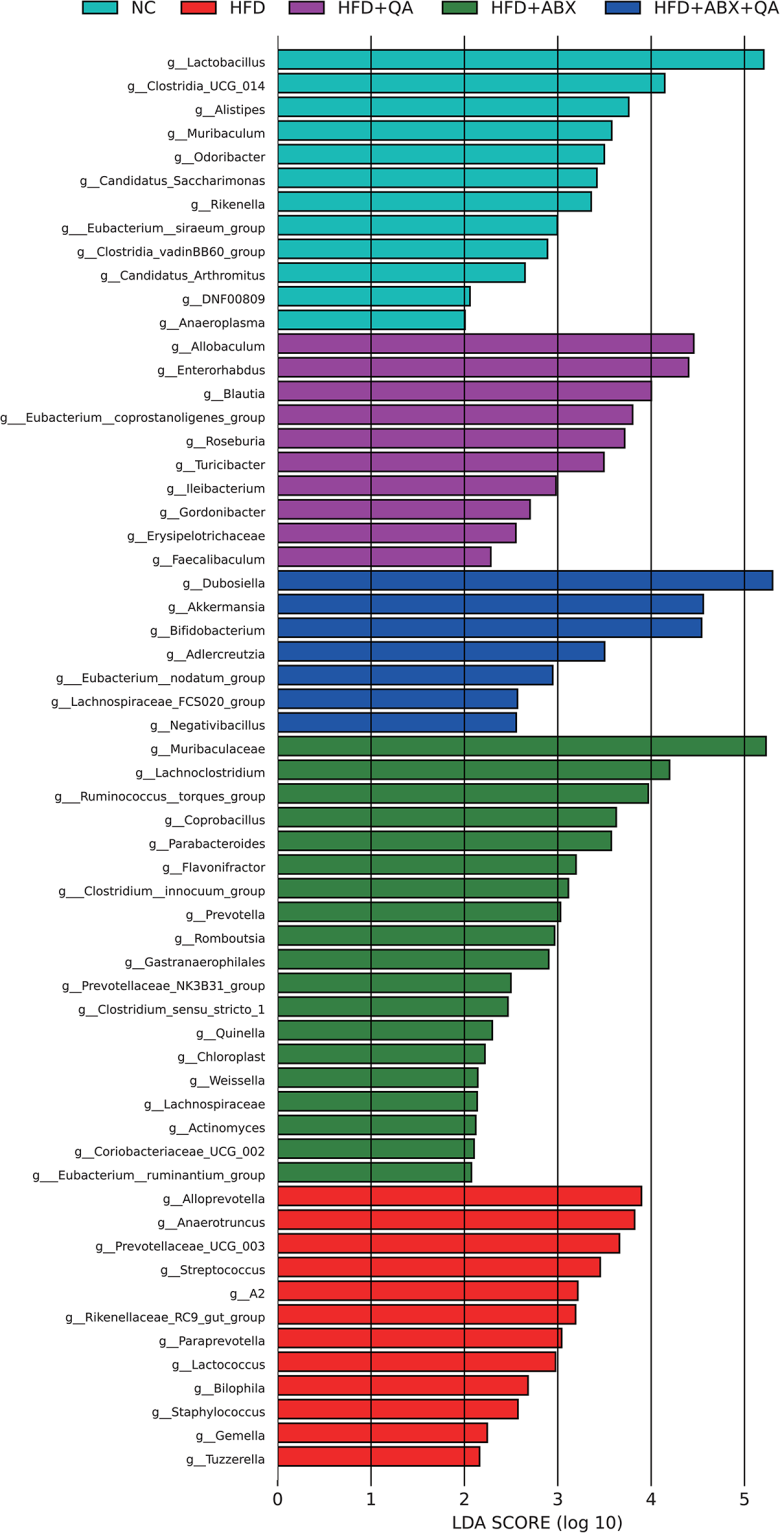
Lefse analysis was applied to analyze the different genus microbiota

Further analysis of genus level microbial found that *Lactobacillus*, *Clostridia_UCG_014*, *Alistipes*, *Muribaculum*, *Odoribacter*, *Candidatus_Saccharimonas*, *Rikenella*, *Eubacterium__siraeum_group*, *Clostridia_vadinBB60_group*, *Candidatus_Arthromitus*, *DNF00809* and *Anaeroplasma* were dominant in NC group (Fig. 4). *Tuzzerella*, *Gemella*, *Staphylococcus*, *Bilophila*, *Lactococcus*, *Paraprevotella*, *Rikenellaceae_RC9_gut_group*, *A2*, *Streptococcus*, *Prevotellaceae_UCG_003*, *Anaerotruncus* and *Alloprevotella* were dominant in HFD group (Fig. 4). *Faecalibaculum*, *Erysipelotrichaceae*, *Gordonibacter*, *Ileibacterium*, *Turicibacter*, *Roseburia*, *Eubacterium__coprostanoligenes_group*, *Blautia*, *Enterorhabdus* and *Allobaculum* were dominant in QA group (Fig. 4). *Muribaculaceae*, *Lachnoclostridium*, *Ruminococcus__torques_group*, *Coprobacillus*, *Parabacteroides*, *Flavonifractor*, *Clostridium__innocuum_group*, *Prevotella*, *Romboutsia*, *Gastranaerophilales*, *Prevotellaceae_NK3B31_group*, *Clostridium_sensu_stricto_1*, *Quinella*, *Chloroplast*, *Weissella*, *Lachnospiraceae*, *Actinomyces*, *Coriobacteriaceae_UCG_002* and *Eubacterium__ruminantium_group* were dominant in ABX group (Fig. 4). *Dubosiella*, *Akkermansia*, *Bifidobacterium*, *Adlercreutzia*, *Eubacterium__nodatum_group*, *Lachnospiraceae_FCS020_group* and *Negativibacillus* were dominant in ABX + QA group (Fig. 4). Combining the above results, it was found that the *Lactobacillus_intestinalis* was the dominant of *Lactobacillus* in the NC group (Fig. 4, Supplementary Fig. 1 and Supplementary Fig. 2). *Streptococcus_danieliae* was the dominant of *Streptococcus* in the HFD group (Fig. 4 and Supplementary Fig. 2). *Ileibacterium_valens* was the dominant of *Ileibacterium* in the QA group (Fig. 4 and Supplementary Fig. 2). *Parabacteroides_gordonii* and *Parabacteroides_goldsteinii* were the dominant of *Parabacteroides* in the ABX group (Fig. 4 and Supplementary Fig. 2). *Bifidobacterium_animalis* was the dominant of *Bifidobacterium* in the ABX + QA group (Fig. 4 and Supplementary Fig. 2). The above results proved that QA improved the gut microbiota disorder by reducing the abundance of *Streptococcus_danieliae*, or promoting the abundance of *Lactobacillus_intestinalis* and *Ileibacterium_valens* in HFD-induced Apoe−/− mice. ABX improved the gut microbiota disorder by promoting the abundance of *Parabacteroides_gordonii* and *Parabacteroides_goldsteinii* in HFD-induced Apoe−/− mice. In addition, the combination of low-frequency ABX and QA may inhibit HFD-induced atherosclerosis in Apoe−/− mice by increasing the *Bifidobacterium_animalis* abundance.

### QA altered serum metabolism in Apoe−/− mice induced by HFD

**Fig. 5:**
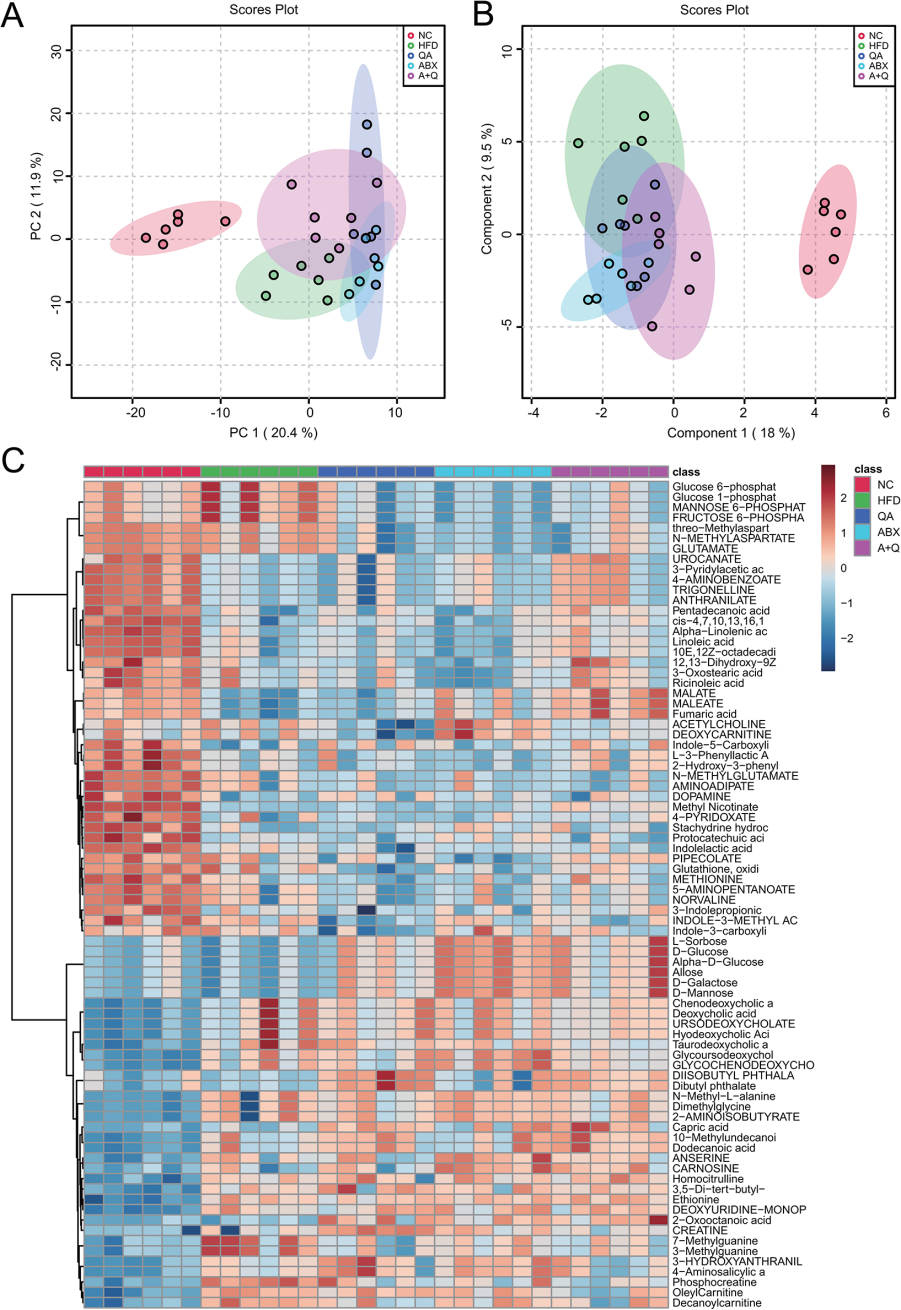
Quinic acid changed serum metabolism of Apoe−/− mice induced by HFD. **(A)** PCA analysis. **(B)** PLSDA analysis. **(C)** Heatmap showed the different metabolites abundance

The intergroup analysis showed that HFD, QA, ABX and ABX + QA groups had some overlap, while NC group deviated, suggesting that HFD induced changes in serum metabolism of Apoe−/− mice (Fig. 5A-B). Analysis of differences between groups showed significant changes in the abundance of 182 metabolites (Fig. 5C). There were 13 metabolites with *P* < 0.001 has been marked red in the heatmap (Fig. 5C). These results indicated that QA changed serum metabolism in Apoe−/− mice induced by HFD.

### QA regulated hepatic lipid metabolism dysfunction in Apoe−/− mice induced by HFD

**Fig. 6:**
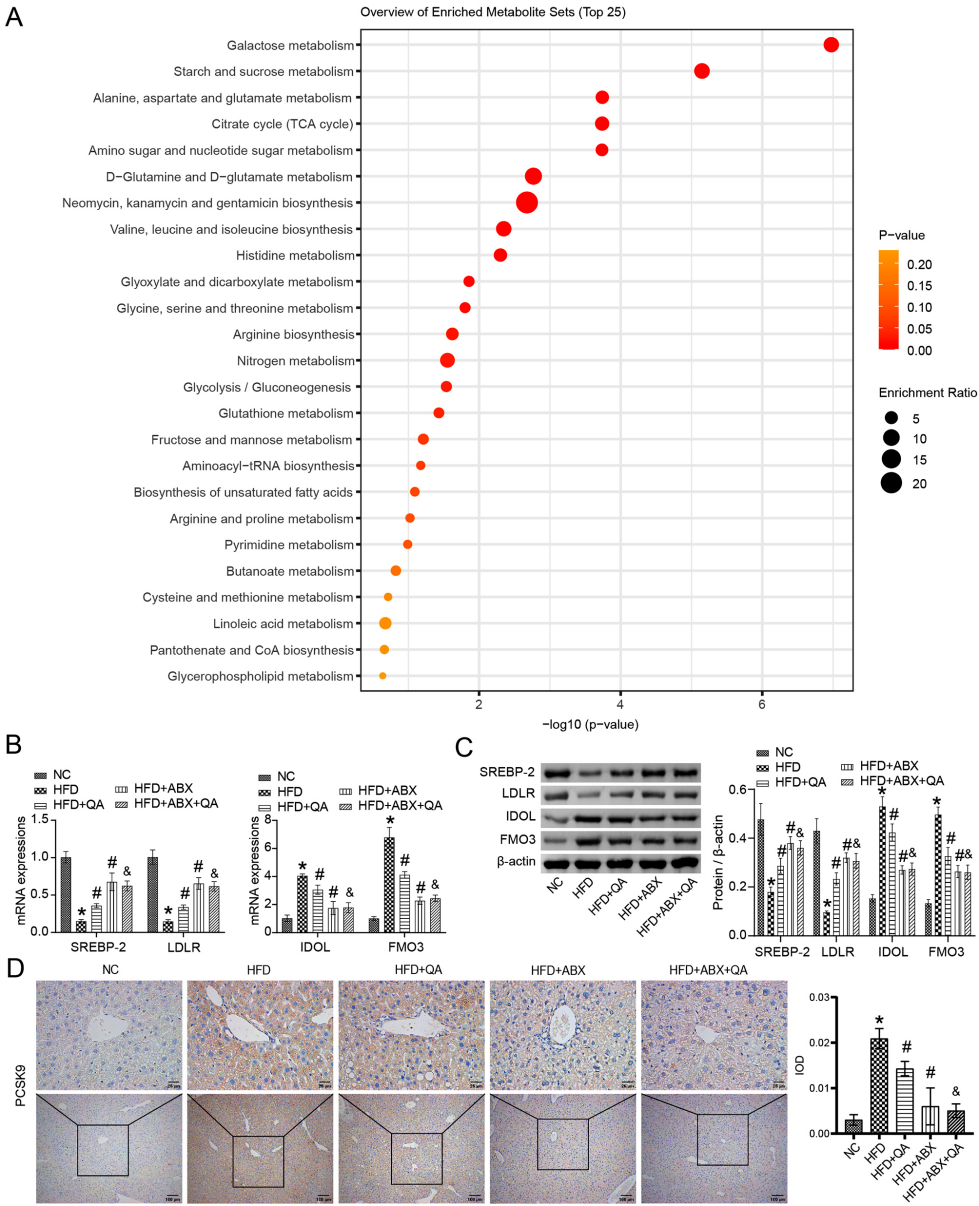
QA improved liver lipid metabolism in Apoe−/− mice induced by HFD. **(A)** All the differential metabolites were enriched in the KEGG database. **(B)** The expressions of SREBP-2, LDLR, IDOL and FMO3 in liver tissues were analyzed by RT-qPCR. **(C)** The expressions of SREBP-2, LDLR, IDOL and FMO3 in liver tissue were analyzed by western blot. **(D)** The expression of PCSK9 in liver tissue was analyzed by IHC. *P < 0.05 vs. NC, #*P* < 0.05 vs. HFD, &*P* < 0.05 vs. HFD + QA

KEGG enrichment showed Galactose metabolism, Starch and sucrose metabolism, Alanine, aspartate and glutamate metabolism, Citrate cycle (TCA cycle) and Amino sugar and nucleotide sugar metabolism were significantly dominant (Fig. 6A). Previous studies have shown that the LXR-IDOL-LDLR feedback [26], the SREBP2/PCSK9 pathway [27], and liver FMO3 expression [28] mediate the liver lipid, cholesterol, and TMAO metabolism levels in atherosclerosis. Our study determined that HFD decreased the expression of SREBP-2 and LDLR, and increased the expression of IDOL, FMO3 and PCSK9 in the liver tissue of Apoe−/− mice (Fig. 6B-D). QA or low-frequency ABX intervention promoted the expression of SREGBP-2 and LDLR, and inhibited the expression of IDOL, FMO3 and PCSK9 in the liver of Apoe−/− mice induced by HFD (Fig. 6B-D). Compared with the QA group, the SREGBP-2 and LDLR expressions were increased, the IDOL, FMO3 and PCSK9 expressions were decreased in QA + ABX group (Fig. 6B-D). However, there was no significant change in SREGBP-2, LDLR, IDOL, FMO3 and PCSK9 levels in the HFD + ABX + QA group compared with the HFD + ABX group (Fig. 6B-D), which proved that low-frequency ABX treatment may promote the effect of QA. These results demonstrated that QA improved liver lipid metabolism in Apoe−/− mice induced by HFD.

### QA regulated the network of microbiota/metabolism in Apoe−/− mice induced by HFD

**Fig. 7:**
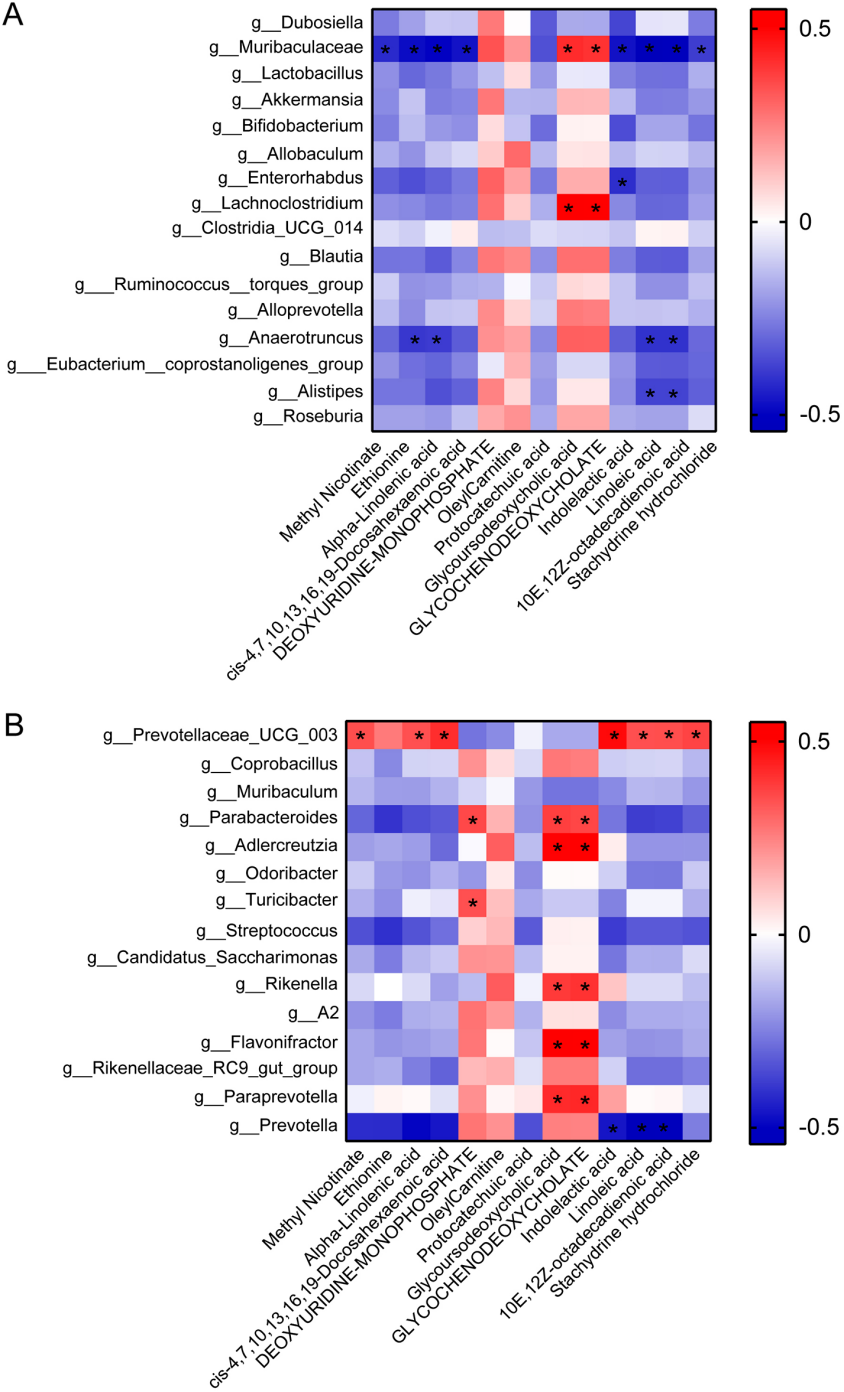
QA regulated the interaction of microbiota and metabolites in Apoe−/− mice induced by HFD. **(A-B)** The correlation of microbiota and metabolites was calculated by Pearson’s coefficient. Red represents positive correlation, blue represents negative correlation. “*” represents *P* < 0.05

To further analyze the interaction between microbiotas and metabolites under the influence of QA, we selected 31 microbiotas (LDA ≥ 3) and 13 metabolites (*P* < 0.001) for correlation analysis (Fig. 7). The results showed that Methyl Nicotinate, cis-4,7,10,13,16,19-Docosahexaenoic acid, and Stachydrine hydrochloride were negatively correlated with *g__Muribaculaceae*, and positively correlated with *g__Prevotellaceae_UCG_003* (Fig. 7A-B). Ethionine was negatively correlated with *g__Muribaculaceae* and *g__Anaerotruncus* (Fig. 7A). Alpha-Linolenic acid was negatively correlated with *g__Muribaculaceae* and *g__Anaerotruncus*, and positively correlated with *g__Prevotellaceae_UCG_003* (Fig. 7A-B). DEOXYURIDINE-MONOPHOSPHATE was positively correlated with *g__Parabacteroides* and *g__Turicibacter* (Fig. 7B). Glycoursodeoxycholic acid and GLYCOCHENODEOXYCHOLATE were positively correlated with *g__Muribaculaceae*, *g__Lachnoclostridium*, *g__Parabacteroides*, *g__Adlercreutzia*, *g__Rikenella*, *g__Flavonifractor*, and *g__Paraprevotella* (Fig. 7A-B). Indolelactic acid was negatively correlated with *g__Muribaculaceae*, *g__Enterorhabdus*, and *g__Prevotella*, and positively correlated with *g__Prevotellaceae_UCG_003* (Fig. 7A-B). Linoleic acid and 10E,12Z-octadecadienoic acid were negatively correlated with *g__Muribaculaceae*, *g__Anaerotruncus*, *g__Alistipes*, and *g__Prevotella*, and positively correlated with *g__Prevotellaceae_UCG_003* (Fig. 7A-B). Based on the above results, it was confirmed that ABX may exert therapeutic effects by regulating DEOXYURIDINE-MONOPHOSPHATE, Glycoursodeoxycholic acid, and GLYCOCHENODEOXYCHOLATE metabolism related to *Parabacteroides* (Fig. 4, Supplementary 2, and Fig. 7). QA treatment may exert therapeutic effects by regulating Indolelactic acid related to *Enterorhabdus* and DEOXYURIDINE-MONOPHOSPHATE related to *Turicibacter* (Figs. 4 and 7). The combination of QA and low-frequency ABX treatment may exert therapeutic effects by regulating Glycoursodeoxycholic acid and GLYCOCHENODEOXYCHOLATE related to *Adlercreutzia* (Figs. 4 and 7). These results demonstrated that the combined treatment of QA and low-frequency ABX could inhibit HFD-induced atherosclerosis in Apoe−/− mice by regulating microbe-related metabolism of Glycoursodeoxycholic acid and GLYCOCHENODEOXYCHOLATE.

### QA improves TMAO-induced inflammatory lesions and endothelial dysfunction in human coronary endothelial cells

**Fig. 8:**
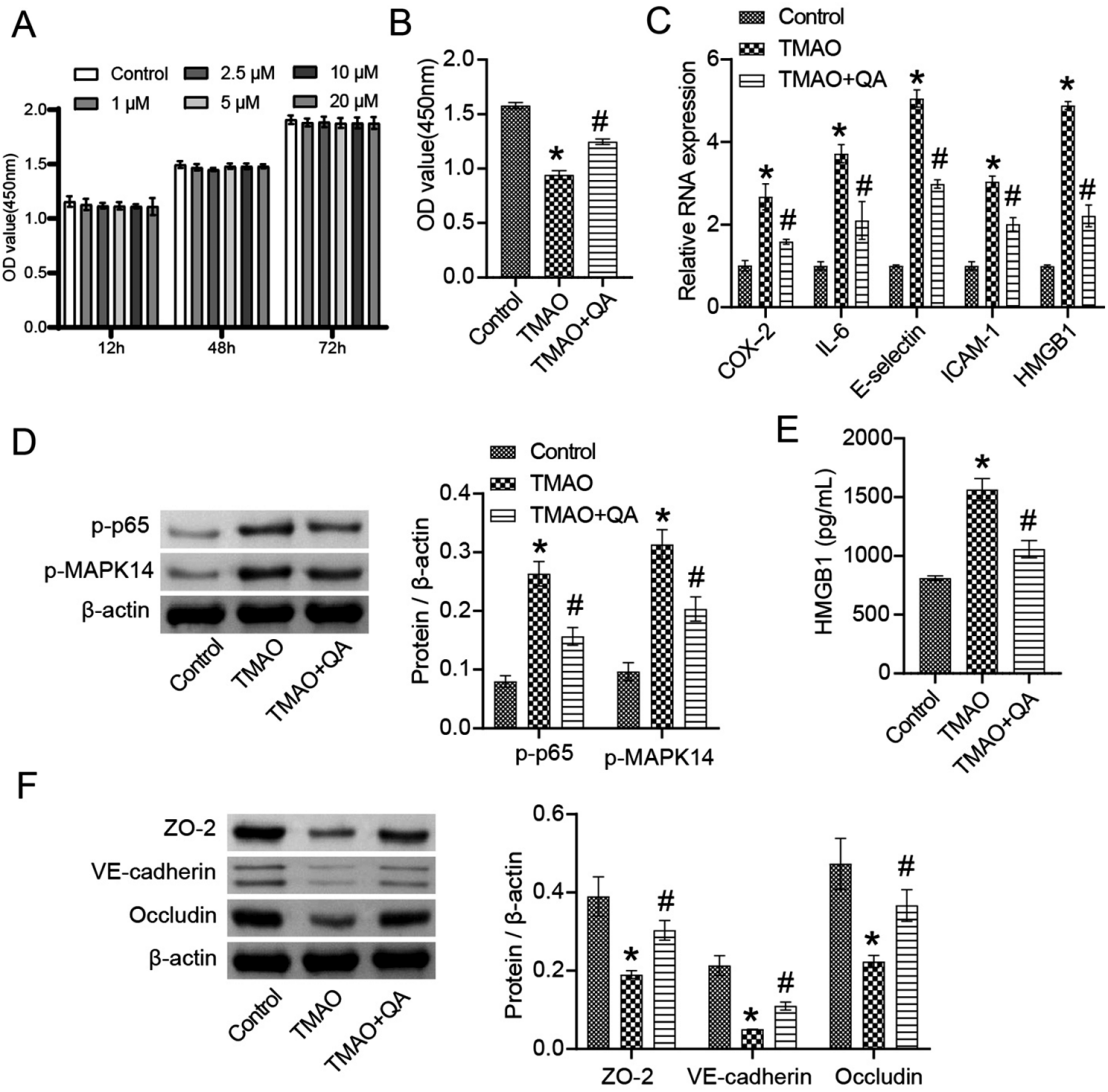
QA improved TMAO-induced inflammatory lesions and endothelial dysfunction in HCAECs. **(A)** CCK-8 was applied to detect the toxicity of QA on HCAECs. **(B)** CCK-8 was used to detect HCAECs proliferation. **(C)** The expression of COX-2, IL-6, E-selectin, ICAM-1, HMGB1 was detected by RT-qPCR. **(D)** The expression of p-P65, p-MAPK14 protein was detected by western blot. **(E)** HMGB1 levels were detected by ELISA. **(F)** The expression of ZO-2, VE-Cadherin and Occludin were detected by western blot. *P < 0.05 vs. Control, #*P* < 0.05 vs. TMAO

We further analyzed the effect of QA on HCAECs cell function in vitro, and CCK-8 showed no significant toxic effect of QA on HCAECs (Fig. 8A). In addition, TMAO inhibited the proliferation of HCAECs, which was reversed by QA (Fig. 8B). TMAO induced increased expression of COX-2, IL-6, E-selectin, ICAM-1, and HMGB1 in HCAECs, and promoted phosphorylation of p-P65 and p-MAPK14 proteins, which was reversed by QA intervention (Fig. 8C-D). TMAO induced the increase of HMGB1 in HCAECs supernatant, and inhibited the expression of ZO-2, VE-Cadherin and Occludin in HCAECs cells (Fig. 8E-F). QA treatment inhibited the expression of HMGB1, and promoted the expression of ZO-2, VE-Cadherin and Occludin in TMAO-induced HCAECs (Fig. 8E-F). These results demonstrated that QA improved TMAO-induced inflammatory lesions and endothelial dysfunction in HCAECs.

### QA improved TMAO-induced LDL trans-endocytosis of HCAECs through HMGB1/SREBP2 axis

**Fig. 9:**
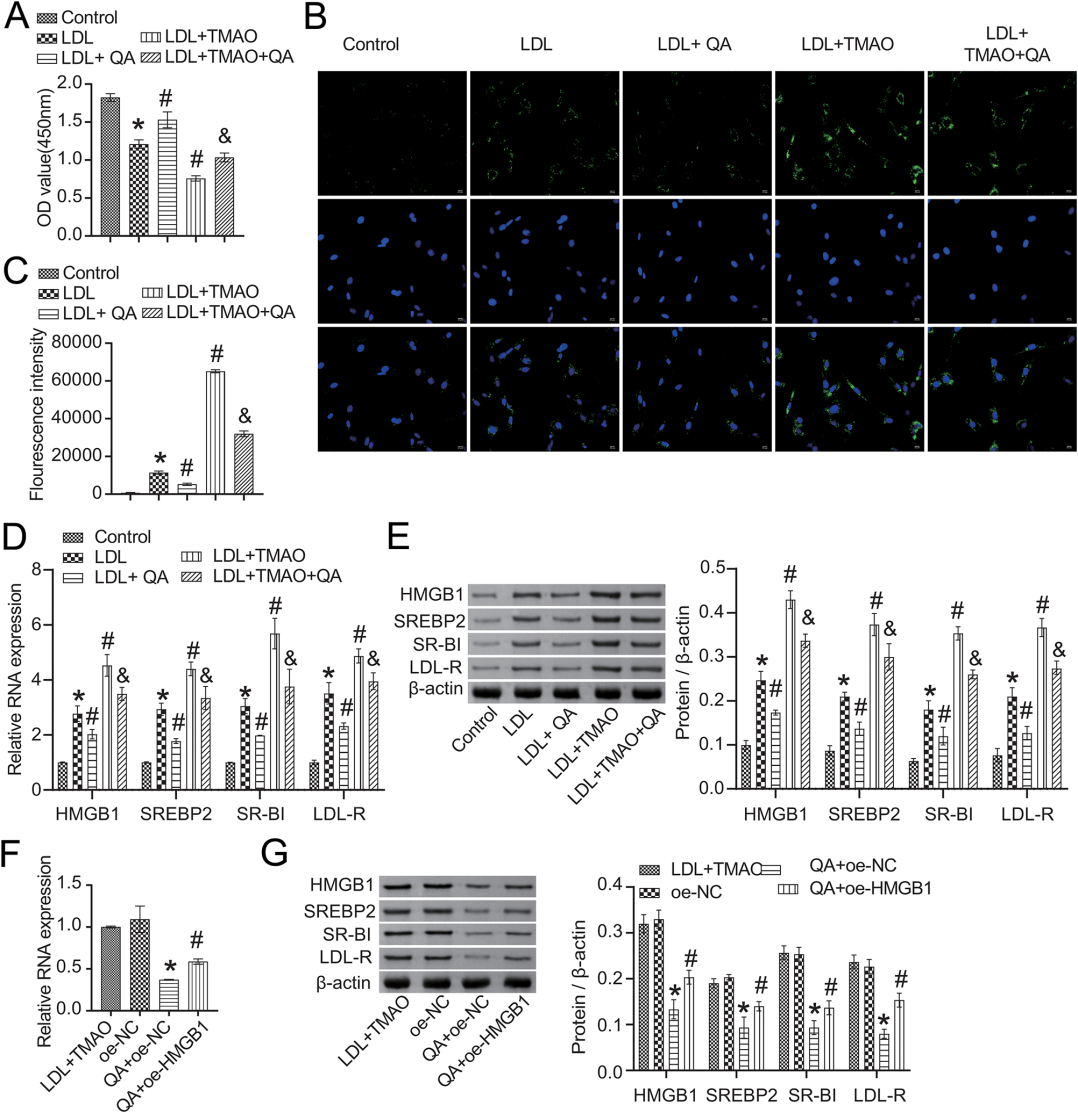
QA improved TMAO-induced LDL trans-endocytosis of HCAECs through HMGB1/SREBP2. **(A)** CCK-8 was applied to detect the proliferation of HCAECs cells. **(B-C)** LDL uptake assay. Scale bar = 25 μm. **(D-G)** The expression of HMGB1, SREBP2, SR-BI and LDL-R was detected by RT-qPCR and western blot. *P < 0.05 vs. Control, #*P* < 0.05 vs. LDL, &*P* < 0.05 vs. LDL + TMAO

We further analyzed the effect of QA on TMAO-induced LDL trans-endocytosis of HCAECs, and found that LDL with or without TMAO inhibited the proliferation of HCAECs (Fig. 9A). QA intervention promoted the proliferation of HCAECs induced by LDL with or without TMAO (Fig. 9A). The LDL endocytosis experiment confirmed that LDL was taken up by HCAECs when LDL was externally added (Fig. 9B-C). At the same time, QA intervention inhibited the uptake of LDL by HCAECs (Fig. 9B-C). TMAO treatment significantly promoted the uptake of LDL by HCAECs, but it was also inhibited by QA intervention (Fig. 9B-C). In addition, the expression of HMGB1, SREBP2, SR-BI and LDL-R in HCAECs was promoted by LDL with or without TMAO, but was inhibited by QA intervention (Fig. 9D-E). It is known that intracellular HMGB1 regulates LDL endocytosis through its nuclear function and SREBP2-SR-BI axis [20]. We further overexpressed HMGB1 on the basis of LDL + TMAO, and the results showed that oe-HMGB1 blocked the inhibitory effect of QA on the expression of HMGB1, SREBP2, SR-BI and LDL-R (Fig. 9F-G). These results demonstrated that QA improved gut microbiota metabolism of TMAO-induced LDL trans-endocytosis of HCAECs by regulating the HMGB1/SREBP2 axis.

## Discussion

The understanding that atherogenesis is an active process rather than a passive cholesterol-storage disease highlights key inflammatory mechanisms [29]. In animal models of NAFLD, HFD increases the accumulation of triglycerides and free fatty acids, and increases the expression of HMGB1 and lipogenesis gene (SREBP-2) in the liver, which is attenuated by the application of Ecklonia cava extract [30]. Sterol regulatory element binding proteins (SREBPs) are major transcription factors that activate the expression of genes associated with cholesterol, fatty acid and triglyceride biosynthesis [31]. We found that HFD induced weight gain and aortic tissue atherogenesis in Apoe−/− mice, but was inhibited by QA or low-frequency ABX treatment. QA inhibits the increase of cholesterol, TMA, TMAO, CXCL13, TIMP-1 and HMGB1 levels in peripheral blood of Apoe−/− mice induced by HFD, suggesting that QA can effectively inhibit HFD-induced atherosclerosis.

Because TMAO and other metabolites of the gut microbiome are associated with platelet overactivity, lipid disorders, and oxidative stress, diet-to-gut microbiome interactions have become an important area of research in the cardiovascular field [32]. Current studies have focused on TMAO prevention strategies, including regulation of the gut microbiome, metabolomics profiles, or probiotic antagonism [33]. The increase of intestinal *Streptococcus_danieliae* exacerbated chronic inflammatory damage in mice with inflammatory skin models [34]. Ileibacterium_valens is helpful in reducing obesity [35] or depression [36, 37], but its role in atherosclerosis is not known. *Bifidobacterium animalis* can act as a probiotic and participate in TMA-TMAO regulation, improve lipid metabolism and reduce atherosclerosis induced by TMAO [38, 39]. Our study found that QA can improve the intestinal flora disorder by reducing the abundance of *Streptococcus_danieliae*, or promoting the abundance of *Lactobacillus_intestinalis* and *Ileibacterium_valens* in HFD-induced Apoe−/− mice. In addition, the combination of low-frequency ABX and QA may inhibit HFD-induced atherosclerosis in Apoe−/− mice by increasing the abundance of *Bifidobacterium_animalis*.

*Lactobacillus intestinalis* is a novel probiotic bacterium that exerts a protective effect against dextran sulfate sodium-induced colitis in mice through the C/EBPA-SAA1/2-Th17 axis [40]. Treatment with *Lactobacillus intestinalis* YT2 significantly alleviates symptoms of menopause, such as increased fat content, decreased bone density, increased pain sensitivity, depressive-like behavior, and cognitive impairments [41]. Arbutin improved intestinal development and inhibited serum lipid level in mice by increasing the *Lactobacillus_intestinalis* abundance [42]. Our research shows that the further enrichment of *Lactobacillus intestinalis* in the QA treated group may be a potential probiotic for improving serum lipid levels in HFD-induced Apoe−/− mice. However, the specific role of *Lactobacillus intestinalis* in QA treatment still needs further investigation.

Through seroepidemiological research, identification of infection factors in human atherosclerotic tissues, and experimental studies demonstrating accelerated atherosclerosis after infection in animal models of atherosclerosis, various bacteria and viral pathogens are associated with atherosclerosis [43]. Multiple evidence suggest a correlation between chronic bacterial infections and atherosclerotic cardiovascular diseases, suggesting that antibiotic treatment may contribute to the secondary prevention of atherosclerosis [44]. However, studies have indicated that the pharmacological disruption of the gut microbiota by antibiotics can exacerbate serum cholesterol and potentially affect cardiovascular diseases [45]. Long-term use of antibiotics (added to drinking water daily for 10 weeks) affects gut bacterial diversity and induces pro-atherogenic metabolic phenotypes, which can be partially reversed by supplementing with tryptophan [46]. Our study confirms that bi-weekly intervention with ABX drinking water for three days for 12 weeks can suppress atherosclerosis induced by a high-fat diet in Apoe-/- mice, contrary to previous studies. However, considering the lower frequency of antibiotic use compared to previous studies [46], this may be the reason antibiotics exert their effects in combating bacterial infections and restoring dysbiosis. That is, high-frequency use of antibiotics may lead to a complete loss of gut bacterial diversity and subsequently have negative effects on the continuum of cardiovascular diseases [47–50]. It is known that the symbiotic Segmented Filamentous Bacteria in the gut and oral cavity are involved in the pathogenesis of TLR2-dependent atherosclerosis through serine dipeptide lipid deposition and arterial wall metabolism [51]. Our study also proved that the combined treatment of QA and low-frequency ABX regulated microbe-related Glycoursodeoxycholic acid and GLYCOCHENODEOXYCHOLATE metabolism in HFD-induced Apoe−/− mice. According to this, we speculate that combining short-term use of antibiotics with targeted drugs against atherosclerosis may be a new therapeutic strategy to improve atherosclerosis induced by HFD in Apoe-/- mice. However, further extensive research is still needed to clarify this.

The gut microbiome has been shown to influence lipid metabolism and lipid levels in blood and tissue in mice and humans [52]. Application of P. tectorius fruit ethanol extract rich in caffeyl quinic acid reduced hyperlipidemia and improved lipid distribution in the liver of hamsters fed a HFD [53]. Galactin-3 (Gal-3) has been shown to be a novel inflammatory agent involved in the processes of endovascular inflammation, lipid endocytosis, macrophage activation, cell proliferation, monocyte chemotaxis, and cell adhesion [54]. The fusion of β-1,4 galactosyltransferase by cigarette smoke or LDL leads to its phosphorylation or activation, the formation of LacCer and the induction of a highly “oxidative stress” environment, resulting in a variety of phenotypes such as inflammation and atherosclerosis [55]. TMAO is formed by the oxidation of TMA by FMO1 and FMO3 [56]. In addition, chronic exposure to galactose containing foods in osteoporotic rats may lead to increased levels of flavin mono-oxygenase-3 (FMO-3) and TMAO [57]. Kanglexin has an effective lipid-lowering effect mediated by activation of AMPK/ SBP-2 /PCSK9/LDLR signaling pathway [58]. Our study demonstrated that QA alters serum Galactose metabolism, promotes SREBP-2 and LDLR, and inhibits the expression of IDOL, FMO3 and PCSK9 in liver tissues of HFD-induced Apoe−/− mice. Its effect may be related to the inhibition of SREBP-2/PCSK9/LDLR signaling pathway and TMA-TMAO transformation.

In addition, SREBP mediated adipogenesis and cholesterol-producing genes, and regulated intestinal liver X-receptor regulatory genes involved in cholesterol transport [59]. TMAO is known to promote nuclear translocation of NF-κB by activating the NF-κB signaling pathway in HCAECs and to act synergically with low-dose pro-atherogenic factors such as HMGB1 [60]. HMGB1 expression is increased in the nucleus and cytoplasm of macrophages and smooth muscle cells in atherosclerotic lesions and is associated with the progression of atherosclerotic plaques [61]. HMGB1 regulated LDL transport in endothelial cells [62]. HMGB1 enhanced the binding of SREBPs to its homologous DNA sequences, and participated in the regulation of the transcription of adipogenic and cholesterol-producing genes [63]. In vivo, we found that QA or ABX treatment inhibited the expression of CAV-1, ABCA1, Mac-3 and SM22α, and promoted the expression of SREBP-1 and LXR in the vascular tissues of HFD-induced Apoe−/− mice, confirming that QA may interfere with the function of vascular endothelial cells. In vitro studies further demonstrated that QA inhibited the phosphorylation of AMPK and NFKB, and the activation of the HMGB1/SREBP2 pathway in TMAO or LDL-induced HCAECs, but was reversed by overexpression of HMGB1. The above studies have demonstrated that QA regulated TMA/TMAO-related lipid metabolism through gut microbiota in gut-liver and inhibited chronic vascular inflammation in atherogenesis of Apoe−/− mice, and the mechanism may be related to the HMGB1/SREBP2 pathway.

The co-localization analysis may be more helpful in elucidating the interactions between atherosclerotic plaques, macrophages, and endothelial cells. However, in this study, due to limitations in experimental techniques, we are unable to perform immunofluorescence staining (Mac or SM22α) on the Oil Red O-stained sections. Unfortunately, due to the completion of animal experiments, there are currently no sufficient samples available to test platelet responsiveness. This is a limitation of our study. Our study preliminarily reveals that QA regulates lipid metabolism and TMA/TMAO metabolism in atherosclerotic mice through the microbiota-metabolite network, which is enhanced by low-frequency (every two weeks) antibiotic treatment. Additionally, QA mediates TMAO-induced LDL trans-endocytosis in HCAECs via the HMGB1/SREBP2 pathway. This study provides new insights into the treatment of atherosclerosis.

## 5 Conclusions

QA regulated the gut-liver lipid metabolism and chronic vascular inflammation of TMA/TMAO through gut microbiota to inhibit the atherogenesis in Apoe−/− mice, which was enhanced by low-frequency (every two weeks) antibiotic treatment. In vitro study reveals that QA mediated TMAO-induced LDL trans-endocytosis in HCAECs via the HMGB1/SREBP2 pathway. This study provided a theoretical basis for the treatment of arteriosclerosis.

### Electronic supplementary material

Below is the link to the electronic supplementary material.

Supplementary Material 1

## Data Availability

The datasets used and analyzed during the current study are available from the corresponding author on reasonable request.

## Abbreviations

QA: Quinic acid
TMA: Trimethylamine
TMAO: Trimethylamine-n-oxide
VSMC: Vascular smooth muscle cell
EAW: Extract
SREBP-2: Lipogenesis gene
FMO-3: Flavin mono-oxygenase-3
