# Supplementary material for: Quinic acid regulated TMA/TMAO-related lipid metabolism and vascular endothelial function through gut microbiota to inhibit atherosclerotic
Source: J Transl Med. 2024 Apr 15;22:352. doi: 10.1186/s12967-024-05120-y (PMC11017595; doi:10.1186/s12967-024-05120-y)

Supplementary Fig. 1. The different microbiota was analyzed by lefse analysis at species level


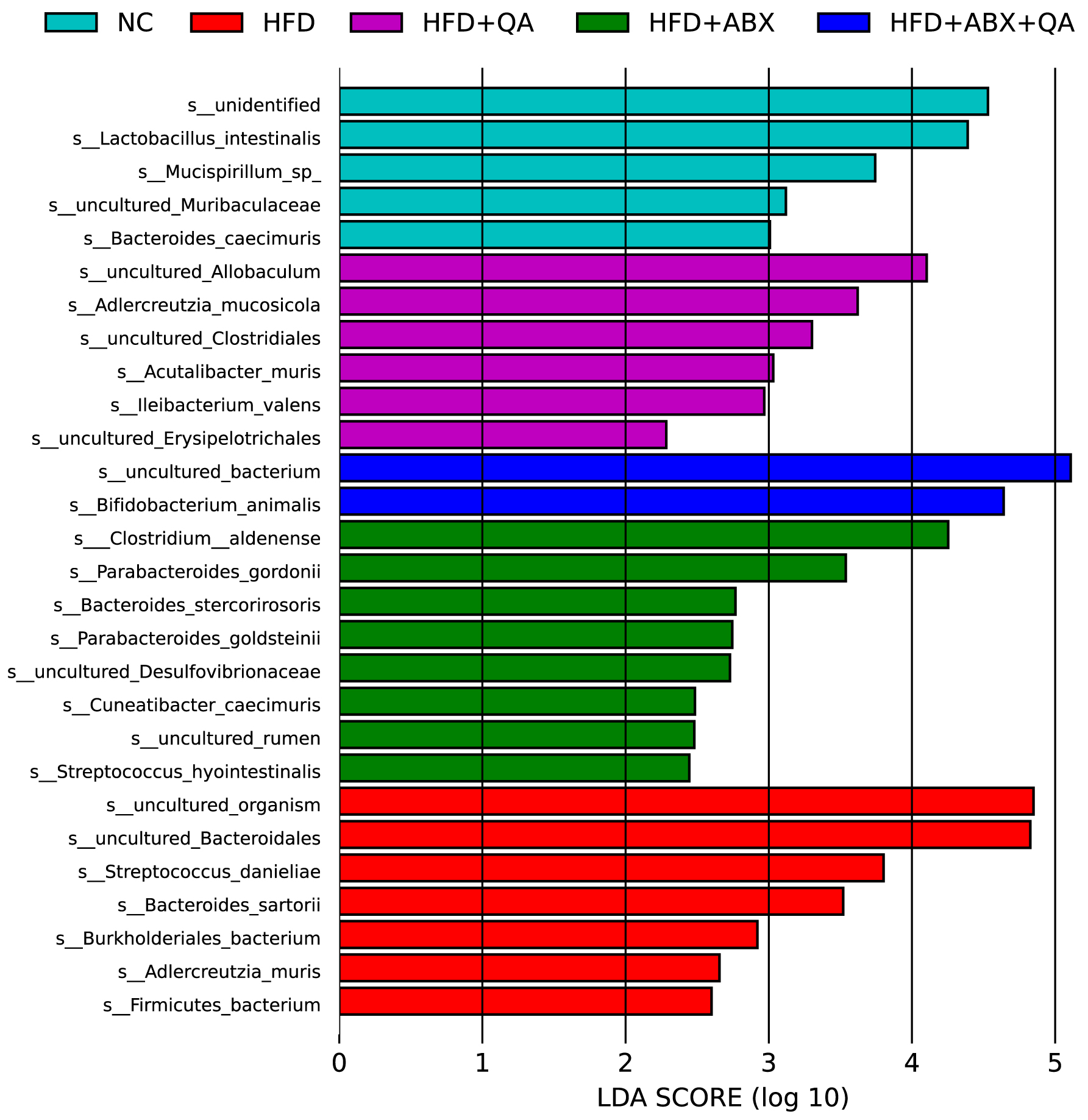


Supplementary Fig. 2. The abundance of species in different groups *P<0.05 vs NC, #P<0.05 vs HFD


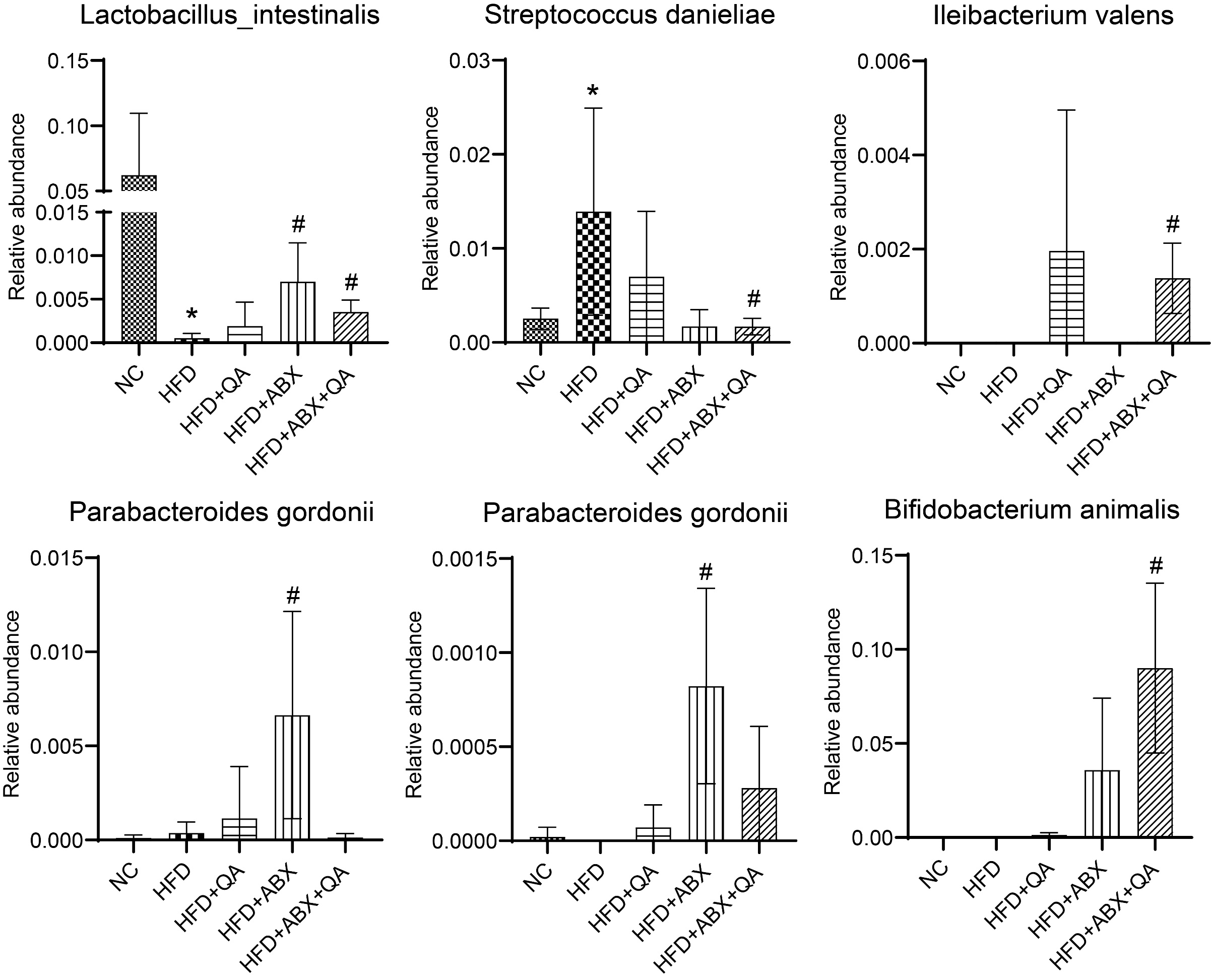

Supplement: Supplementary file 1 — Supplementary Material 1 [file 12967_2024_5120_MOESM1_ESM.docx]
